# ORGANIZATIONAL AND OLDER ADULT VOLUNTEER PERSPECTIVES ON ROLE CONFLICT MANAGEMENT STRATEGIES

**DOI:** 10.1093/geroni/igz038.3163

**Published:** 2019-11-08

**Authors:** Jennifer A Crittenden, Sandra S Butler

**Affiliations:** 1 UMaine Center on Aging, University of Maine, Bangor, Maine, United States; 2 University of Maine, Orono, Maine, United States

## Abstract

Older adults are increasingly occupying multiple life roles, including working, caregiving, and volunteering, creating the opportunity for role conflict. Such conflict occurs when stress and strain created by the demands of multiple life roles outstrips an individual’s resources to successfully manage such demands. A two-phase research study was recently completed with 1,697 RSVP volunteers drawn from 55 RSVP program sites across the country (Phase I) with a follow-up survey of RSVP programs conducted with 17 sites (Phase II). Grounded in role theory, the Phase I volunteer survey explored role conflict in addition to self-reported strategies used to mitigate the experience of role conflict. The Phase II program survey gathered responses from volunteer managers and staff about the strategies used by their older adult volunteers to avoid and address role conflict. Based on findings from both surveys, caregivers engaged the following strategies in order to minimize role conflict: obtaining respite care, and volunteering alongside their care recipient. Worker-specific strategies focused largely on time management and included volunteering during off-work hours and completing time-limited volunteer assignments. While a high level of convergence was noted between volunteer manager and volunteer perspectives, two themes emerged from the volunteer survey that were not identified in program survey responses: seeking volunteer opportunities that leverage similar skills and experiences across roles and seeking volunteer opportunities that provide a different experience from that of other roles. Implications for future research and volunteer management strategies will be discussed.

